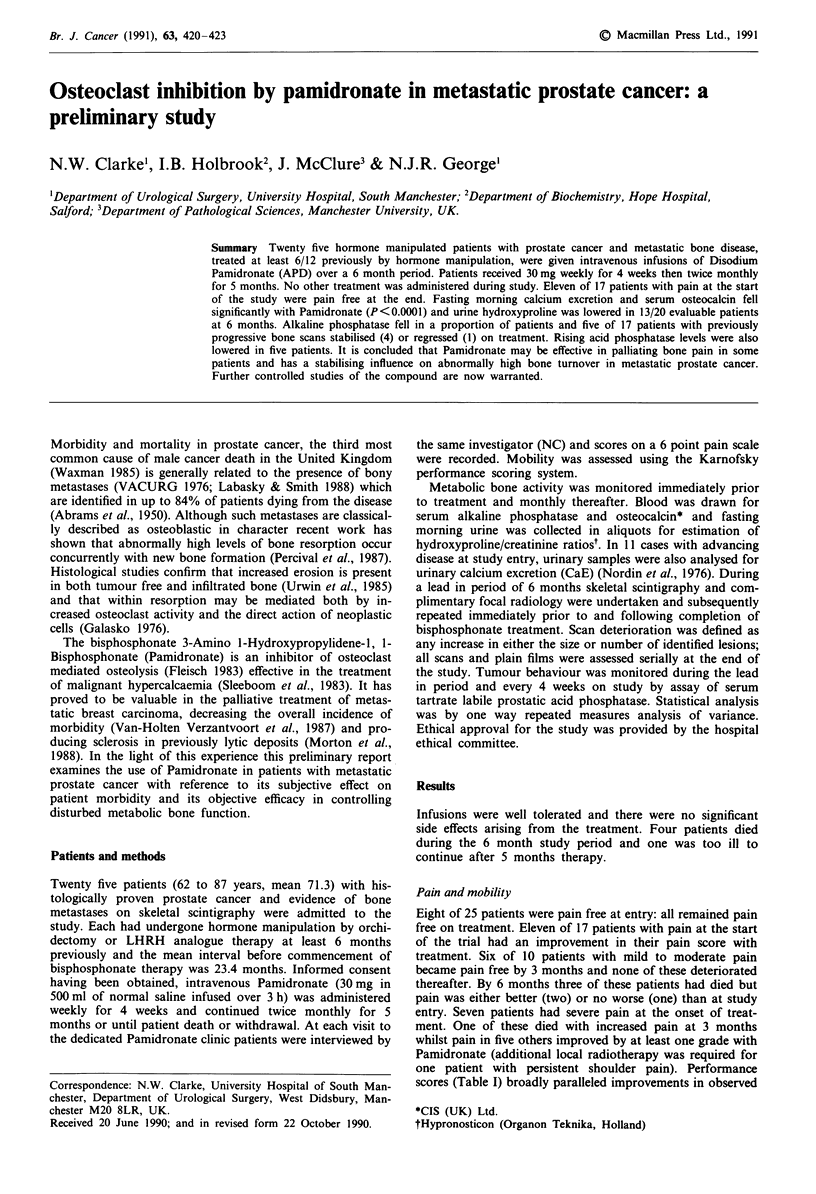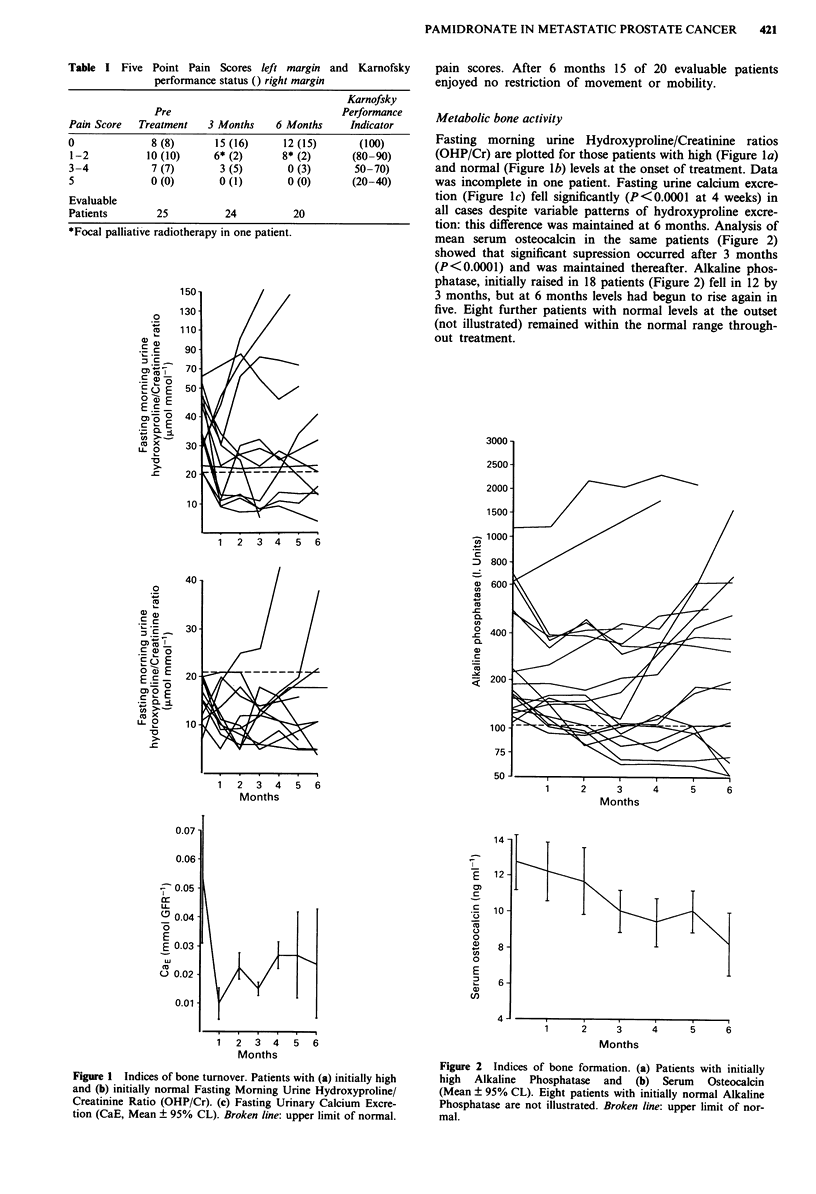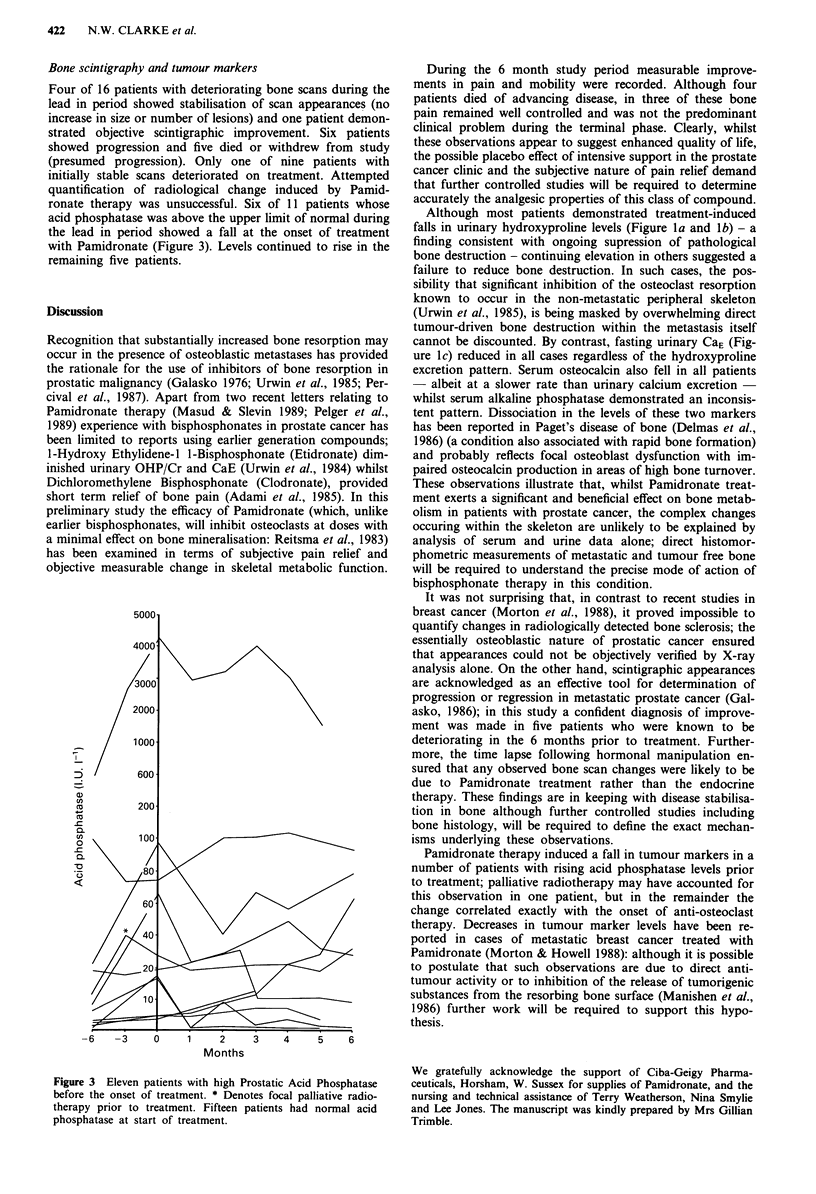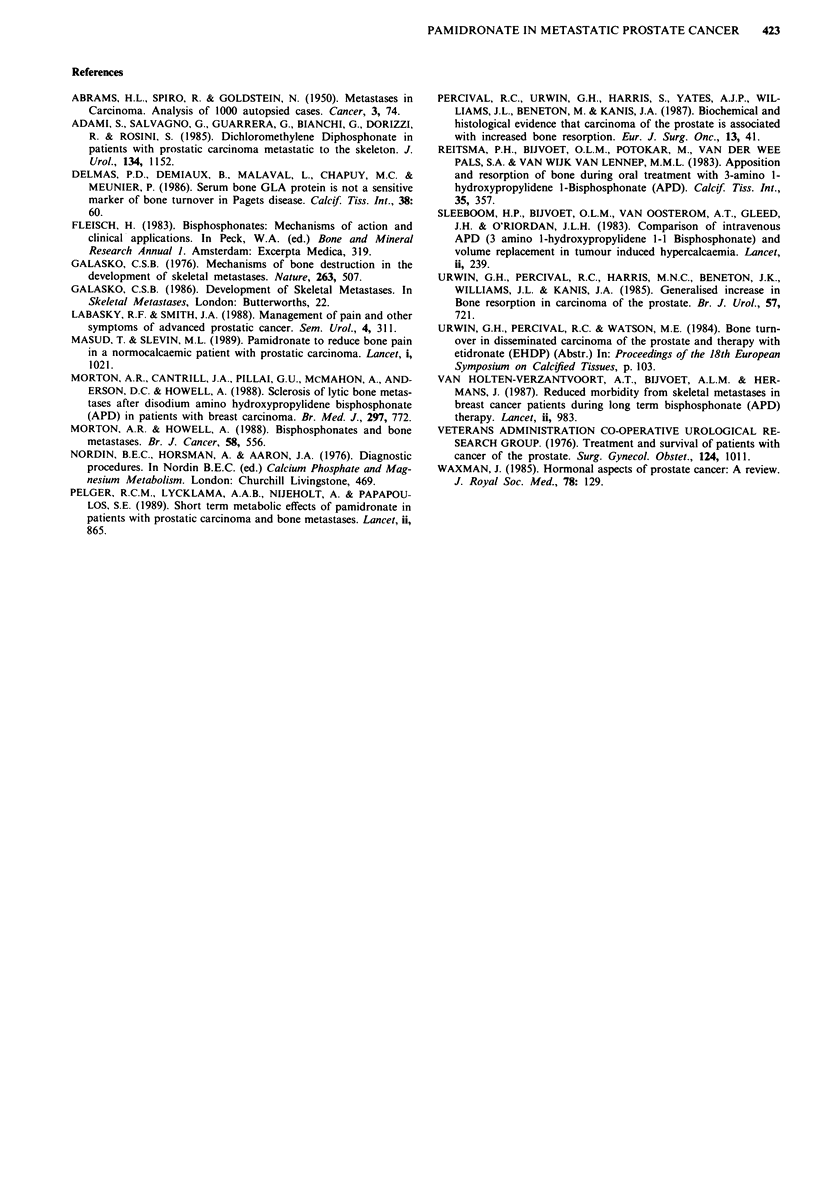# Osteoclast inhibition by pamidronate in metastatic prostate cancer: a preliminary study.

**DOI:** 10.1038/bjc.1991.97

**Published:** 1991-03

**Authors:** N. W. Clarke, I. B. Holbrook, J. McClure, N. J. George

**Affiliations:** Department of Urological Surgery, University Hospital, South Manchester, UK.

## Abstract

Twenty five hormone manipulated patients with prostate cancer and metastatic bone disease, treated at least 6/12 previously by hormone manipulation, were given intravenous infusions of Disodium Pamidronate (APD) over a 6 month period. Patients received 30 mg weekly for 4 weeks then twice monthly for 5 months. No other treatment was administered during study. Eleven of 17 patients with pain at the start of the study were pain free at the end. Fasting morning calcium excretion and serum osteocalcin fell significantly with Pamidronate (P less than 0.0001) and urine hydroxyproline was lowered in 13/20 evaluable patients at 6 months. Alkaline phosphatase fell in a proportion of patients and five of 17 patients with previously progressive bone scans stabilised (4) or regressed (1) on treatment. Rising acid phosphatase levels were also lowered in five patients. It is concluded that Pamidronate may be effective in palliating bone pain in some patients and has a stabilising influence on abnormally high bone turnover in metastatic prostate cancer. Further controlled studies of the compound are now warranted.


Br. J. Cancer (1991), 63, 420-423 © Macmillan Press Ltd., 1991

### Osteoclast inhibition by pamidronate in metastatic prostate cancer: a preliminary study

N.W. Clarke[1], I.B. Holbrook[2], J. McClure[3] & N.J.R. George[1]

[1]*Department of Urological Surgery, University Hospital, South Manchester;* [2]*Department of Biochemistry, Hope Hospital, Salford;* [3]*Department of Pathological Sciences, Manchester University, UK.*

**Summary** Twenty five hormone manipulated patients with prostate cancer and metastatic bone disease, treated at least 6/12 previously by hormone manipulation, were given intravenous infusions of Disodium Pamidronate (APD) over a 6 month period. Patients received 30 mg weekly for 4 weeks then twice monthly for 5 months. No other treatment was administered during study. Eleven of 17 patients with pain at the start of the study were pain free at the end. Fasting morning calcium excretion and serum osteocalcin fell significantly with Pamidronate ($P < 0.0001$) and urine hydroxyproline was lowered in 13/20 evaluable patients at 6 months. Alkaline phosphatase fell in a proportion of patients and five of 17 patients with previously progressive bone scans stabilised (4) or regressed (1) on treatment. Rising acid phosphatase levels were also lowered in five patients. It is concluded that Pamidronate may be effective in palliating bone pain in some patients and has a stabilising influence on abnormally high bone turnover in metastatic prostate cancer. Further controlled studies of the compound are now warranted.

Morbidity and mortality in prostate cancer, the third most common cause of male cancer death in the United Kingdom (Waxman 1985) is generally related to the presence of bony metastases (VACURG 1976; Labasky & Smith 1988) which are identified in up to 84% of patients dying from the disease (Abrams *et al.*, 1950). Although such metastases are classically described as osteoblastic in character recent work has shown that abnormally high levels of bone resorption occur concurrently with new bone formation (Percival *et al.*, 1987). Histological studies confirm that increased erosion is present in both tumour free and infiltrated bone (Urwin *et al.*, 1985) and that within resorption may be mediated both by increased osteoclast activity and the direct action of neoplastic cells (Galasko 1976).

The bisphosphonate 3-Amino 1-Hydroxypropylidene-1, 1-Bisphosphonate (Pamidronate) is an inhibitor of osteoclast mediated osteolysis (Fleisch 1983) effective in the treatment of malignant hypercalcaemia (Sleeboom *et al.*, 1983). It has proved to be valuable in the palliative treatment of metastatic breast carcinoma, decreasing the overall incidence of morbidity (Van-Holten Verzantvoort *et al.*, 1987) and producing sclerosis in previously lytic deposits (Morton *et al.*, 1988). In the light of this experience this preliminary report examines the use of Pamidronate in patients with metastatic prostate cancer with reference to its subjective effect on patient morbidity and its objective efficacy in controlling disturbed metabolic bone function.

### Patients and methods

Twenty five patients (62 to 87 years, mean 71.3) with histologically proven prostate cancer and evidence of bone metastases on skeletal scintigraphy were admitted to the study. Each had undergone hormone manipulation by orchidectomy or LHRH analogue therapy at least 6 months previously and the mean interval before commencement of bisphosphonate therapy was 23.4 months. Informed consent having been obtained, intravenous Pamidronate (30 mg in 500 ml of normal saline infused over 3 h) was administered weekly for 4 weeks and continued twice monthly for 5 months or until patient death or withdrawal. At each visit to the dedicated Pamidronate clinic patients were interviewed by the same investigator (NC) and scores on a 6 point pain scale were recorded. Mobility was assessed using the Karnofsky performance scoring system.

Metabolic bone activity was monitored immediately prior to treatment and monthly thereafter. Blood was drawn for serum alkaline phosphatase and osteocalcin* and fasting morning urine was collected in aliquots for estimation of hydroxyproline/creatinine ratios†. In 11 cases with advancing disease at study entry, urinary samples were also analysed for urinary calcium excretion (CaE) (Nordin *et al.*, 1976). During a lead in period of 6 months skeletal scintigraphy and complimentary focal radiology were undertaken and subsequently repeated immediately prior to and following completion of bisphosphonate treatment. Scan deterioration was defined as any increase in either the size or number of identified lesions; all scans and plain films were assessed serially at the end of the study. Tumour behaviour was monitored during the lead in period and every 4 weeks on study by assay of serum tartrate labile prostatic acid phosphatase. Statistical analysis was by one way repeated measures analysis of variance. Ethical approval for the study was provided by the hospital ethical committee.

### Results

Infusions were well tolerated and there were no significant side effects arising from the treatment. Four patients died during the 6 month study period and one was too ill to continue after 5 months therapy.

### *Pain and mobility*

Eight of 25 patients were pain free at entry: all remained pain free on treatment. Eleven of 17 patients with pain at the start of the trial had an improvement in their pain score with treatment. Six of 10 patients with mild to moderate pain became pain free by 3 months and none of these deteriorated thereafter. By 6 months three of these patients had died but pain was either better (two) or no worse (one) than at study entry. Seven patients had severe pain at the onset of treatment. One of these died with increased pain at 3 months whilst pain in five others improved by at least one grade with Pamidronate (additional local radiotherapy was required for one patient with persistent shoulder pain). Performance scores (Table I) broadly paralleled improvements in observed

Correspondence: N.W. Clarke, University Hospital of South Manchester, Department of Urological Surgery, West Didsbury, Manchester M20 8LR, UK.
Received 20 June 1990; and in revised form 22 October 1990.

*CIS (UK) Ltd.
†Hypronosticon (Organon Teknika, Holland)

**Table I** Five Point Pain Scores *left margin* and Karnofsky performance status () *right margin*

| *Pain Score* | *Pre Treatment* | *3 Months* | *6 Months* | *Karnofsky Performance Indicator* |
|---|---|---|---|---|
| 0 | 8 (8) | 15 (16) | 12 (15) | (100) |
| 1-2 | 10 (10) | 6* (2) | 8* (2) | (80-90) |
| 3-4 | 7 (7) | 3 (5) | 0 (3) | 50-70) |
| 5 | 0 (0) | 0 (1) | 0 (0) | (20-40) |
| Evaluable Patients | 25 | 24 | 20 | |

*Focal palliative radiotherapy in one patient.

**Figure 1** Indices of bone turnover. Patients with (**a**) initially high and (**b**) initially normal Fasting Morning Urine Hydroxyproline/Creatinine Ratio (OHP/Cr). (**c**) Fasting Urinary Calcium Excretion (CaE, Mean ± 95% CL). *Broken line*: upper limit of normal.

pain scores. After 6 months 15 of 20 evaluable patients enjoyed no restriction of movement or mobility.

*Metabolic bone activity*

Fasting morning urine Hydroxyproline/Creatinine ratios (OHP/Cr) are plotted for those patients with high (Figure 1*a*) and normal (Figure 1*b*) levels at the onset of treatment. Data was incomplete in one patient. Fasting urine calcium excretion (Figure 1*c*) fell significantly ($P < 0.0001$ at 4 weeks) in all cases despite variable patterns of hydroxyproline excretion: this difference was maintained at 6 months. Analysis of mean serum osteocalcin in the same patients (Figure 2) showed that significant supression occurred after 3 months ($P < 0.0001$) and was maintained thereafter. Alkaline phosphatase, initially raised in 18 patients (Figure 2) fell in 12 by 3 months, but at 6 months levels had begun to rise again in five. Eight further patients with normal levels at the outset (not illustrated) remained within the normal range throughout treatment.

**Figure 2** Indices of bone formation. (**a**) Patients with initially high Alkaline Phosphatase and (**b**) Serum Osteocalcin (Mean ± 95% CL). Eight patients with initially normal Alkaline Phosphatase are not illustrated. *Broken line*: upper limit of normal.

*Bone scintigraphy and tumour markers*

Four of 16 patients with deteriorating bone scans during the lead in period showed stabilisation of scan appearances (no increase in size or number of lesions) and one patient demonstrated objective scintigraphic improvement. Six patients showed progression and five died or withdrew from study (presumed progression). Only one of nine patients with initially stable scans deteriorated on treatment. Attempted quantification of radiological change induced by Pamidronate therapy was unsuccessful. Six of 11 patients whose acid phosphatase was above the upper limit of normal during the lead in period showed a fall at the onset of treatment with Pamidronate (Figure 3). Levels continued to rise in the remaining five patients.

### Discussion

Recognition that substantially increased bone resorption may occur in the presence of osteoblastic metastases has provided the rationale for the use of inhibitors of bone resorption in prostatic malignancy (Galasko 1976; Urwin *et al.*, 1985; Percival *et al.*, 1987). Apart from two recent letters relating to Pamidronate therapy (Masud & Slevin 1989; Pelger *et al.*, 1989) experience with bisphosphonates in prostate cancer has been limited to reports using earlier generation compounds; 1-Hydroxy Ethylidene-1 1-Bisphosphonate (Etidronate) diminished urinary OHP/Cr and CaE (Urwin *et al.*, 1984) whilst Dichloromethylene Bisphosphonate (Clodronate), provided short term relief of bone pain (Adami *et al.*, 1985). In this preliminary study the efficacy of Pamidronate (which, unlike earlier bisphosphonates, will inhibit osteoclasts at doses with a minimal effect on bone mineralisation: Reitsma *et al.*, 1983) has been examined in terms of subjective pain relief and objective measurable change in skeletal metabolic function.

**Figure 3** Eleven patients with high Prostatic Acid Phosphatase before the onset of treatment. * Denotes focal palliative radiotherapy prior to treatment. Fifteen patients had normal acid phosphatase at start of treatment.

During the 6 month study period measurable improvements in pain and mobility were recorded. Although four patients died of advancing disease, in three of these bone pain remained well controlled and was not the predominant clinical problem during the terminal phase. Clearly, whilst these observations appear to suggest enhanced quality of life, the possible placebo effect of intensive support in the prostate cancer clinic and the subjective nature of pain relief demand that further controlled studies will be required to determine accurately the analgesic properties of this class of compound.

Although most patients demonstrated treatment-induced falls in urinary hydroxyproline levels (Figure 1*a* and 1*b*) – a finding consistent with ongoing supression of pathological bone destruction – continuing elevation in others suggested a failure to reduce bone destruction. In such cases, the possibility that significant inhibition of the osteoclast resorption known to occur in the non-metastatic peripheral skeleton (Urwin *et al.*, 1985), is being masked by overwhelming direct tumour-driven bone destruction within the metastasis itself cannot be discounted. By contrast, fasting urinary $Ca_E$ (Figure 1*c*) reduced in all cases regardless of the hydroxyproline excretion pattern. Serum osteocalcin also fell in all patients — albeit at a slower rate than urinary calcium excretion — whilst serum alkaline phosphatase demonstrated an inconsistent pattern. Dissociation in the levels of these two markers has been reported in Paget's disease of bone (Delmas *et al.*, 1986) (a condition also associated with rapid bone formation) and probably reflects focal osteoblast dysfunction with impaired osteocalcin production in areas of high bone turnover. These observations illustrate that, whilst Pamidronate treatment exerts a significant and beneficial effect on bone metabolism in patients with prostate cancer, the complex changes occuring within the skeleton are unlikely to be explained by analysis of serum and urine data alone; direct histomorphometric measurements of metastatic and tumour free bone will be required to understand the precise mode of action of bisphosphonate therapy in this condition.

It was not surprising that, in contrast to recent studies in breast cancer (Morton *et al.*, 1988), it proved impossible to quantify changes in radiologically detected bone sclerosis; the essentially osteoblastic nature of prostatic cancer ensured that appearances could not be objectively verified by X-ray analysis alone. On the other hand, scintigraphic appearances are acknowledged as an effective tool for determination of progression or regression in metastatic prostate cancer (Galasko, 1986); in this study a confident diagnosis of improvement was made in five patients who were known to be deteriorating in the 6 months prior to treatment. Furthermore, the time lapse following hormonal manipulation ensured that any observed bone scan changes were likely to be due to Pamidronate treatment rather than the endocrine therapy. These findings are in keeping with disease stabilisation in bone although further controlled studies including bone histology, will be required to define the exact mechanisms underlying these observations.

Pamidronate therapy induced a fall in tumour markers in a number of patients with rising acid phosphatase levels prior to treatment; palliative radiotherapy may have accounted for this observation in one patient, but in the remainder the change correlated exactly with the onset of anti-osteoclast therapy. Decreases in tumour marker levels have been reported in cases of metastatic breast cancer treated with Pamidronate (Morton & Howell 1988): although it is possible to postulate that such observations are due to direct anti-tumour activity or to inhibition of the release of tumorigenic substances from the resorbing bone surface (Manishen *et al.*, 1986) further work will be required to support this hypothesis.

We gratefully acknowledge the support of Ciba-Geigy Pharmaceuticals, Horsham, W. Sussex for supplies of Pamidronate, and the nursing and technical assistance of Terry Weatherson, Nina Smylie and Lee Jones. The manuscript was kindly prepared by Mrs Gillian Trimble.

### References

ABRAMS, H.L., SPIRO, R. & GOLDSTEIN, N. (1950). Metastases in Carcinoma. Analysis of 1000 autopsied cases. *Cancer*, **3**, 74.

ADAMI, S., SALVAGNO, G., GUARRERA, G., BIANCHI, G., DORIZZI, R. & ROSINI, S. (1985). Dichloromethylene Diphosphonate in patients with prostatic carcinoma metastatic to the skeleton. *J. Urol.*, **134**, 1152.

DELMAS, P.D., DEMIAUX, B., MALAVAL, L., CHAPUY, M.C. & MEUNIER, P. (1986). Serum bone GLA protein is not a sensitive marker of bone turnover in Pagets disease. *Calcif. Tiss. Int.*, **38**: 60.

FLEISCH, H. (1983). Bisphosphonates: Mechanisms of action and clinical applications. In Peck, W.A. (ed.) *Bone and Mineral Research Annual 1*. Amsterdam: Excerpta Medica, 319.

GALASKO, C.S.B. (1976). Mechanisms of bone destruction in the development of skeletal metastases. *Nature*, **263**, 507.

GALASKO, C.S.B. (1986). Development of Skeletal Metastases. In *Skeletal Metastases*, London: Butterworths, 22.

LABASKY, R.F. & SMITH, J.A. (1988). Management of pain and other symptoms of advanced prostatic cancer. *Sem. Urol.*, **4**, 311.

MASUD, T. & SLEVIN, M.L. (1989). Pamidronate to reduce bone pain in a normocalcaemic patient with prostatic carcinoma. *Lancet*, **i**, 1021.

MORTON, A.R., CANTRILL, J.A., PILLAI, G.U., MCMAHON, A., ANDERSON, D.C. & HOWELL, A. (1988). Sclerosis of lytic bone metastases after disodium amino hydroxypropylidene bisphosphonate (APD) in patients with breast carcinoma. *Br. Med. J.*, **297**, 772.

MORTON, A.R. & HOWELL, A. (1988). Bisphosphonates and bone metastases. *Br. J. Cancer*, **58**, 556.

NORDIN, B.E.C., HORSMAN, A. & AARON, J.A. (1976). Diagnostic procedures. In Nordin B.E.C. (ed.) *Calcium Phosphate and Magnesium Metabolism*. London: Churchill Livingstone, 469.

PELGER, R.C.M., LYCKLAMA, A.A.B., NIJEHOLT, A. & PAPAPOULOS, S.E. (1989). Short term metabolic effects of pamidronate in patients with prostatic carcinoma and bone metastases. *Lancet*, **ii**, 865.

PERCIVAL, R.C., URWIN, G.H., HARRIS, S., YATES, A.J.P., WILLIAMS, J.L., BENETON, M. & KANIS, J.A. (1987). Biochemical and histological evidence that carcinoma of the prostate is associated with increased bone resorption. *Eur. J. Surg. Onc.*, **13**, 41.

REITSMA, P.H., BIJVOET, O.L.M., POTOKAR, M., VAN DER WEE PALS, S.A. & VAN WIJK VAN LENNEP, M.M.L. (1983). Apposition and resorption of bone during oral treatment with 3-amino 1-hydroxypropylidene 1-Bisphosphonate (APD). *Calcif. Tiss. Int.*, **35**, 357.

SLEEBOOM, H.P., BIJVOET, O.L.M., VAN OOSTEROM, A.T., GLEED, J.H. & O'RIORDAN, J.L.H. (1983). Comparison of intravenous APD (3 amino 1-hydroxypropylidene 1-1 Bisphosphonate) and volume replacement in tumour induced hypercalcaemia. *Lancet*, **ii**, 239.

URWIN, G.H., PERCIVAL, R.C., HARRIS, M.N.C., BENETON, J.K., WILLIAMS, J.L. & KANIS, J.A. (1985). Generalised increase in Bone resorption in carcinoma of the prostate. *Br. J. Urol.*, **57**, 721.

URWIN, G.H., PERCIVAL, R.C. & WATSON, M.E. (1984). Bone turnover in disseminated carcinoma of the prostate and therapy with etidronate (EHDP) (Abstr.) In: *Proceedings of the 18th European Symposium on Calcified Tissues*, p. 103.

VAN HOLTEN-VERZANTVOORT, A.T., BIJVOET, A.L.M. & HERMANS, J. (1987). Reduced morbidity from skeletal metastases in breast cancer patients during long term bisphosphonate (APD) therapy. *Lancet*, **ii**, 983.

VETERANS ADMINISTRATION CO-OPERATIVE UROLOGICAL RESEARCH GROUP. (1976). Treatment and survival of patients with cancer of the prostate. *Surg. Gynecol. Obstet.*, **124**, 1011.

WAXMAN, J. (1985). Hormonal aspects of prostate cancer: A review. *J. Royal Soc. Med.*, **78**: 129.